# Evaluation of the Anti-Inflammatory Activity of Selected Plant Extracts in an In Vitro Model of Inflammation Using LPS-Stimulated Macrophages

**DOI:** 10.3390/biomedicines14051174

**Published:** 2026-05-21

**Authors:** Karolina Merecz, Kinga Suska, Olga Biniszewska, Mikołaj Hirsa, Aneta Wojdyło, Aleksandra Tarasiuk-Zawadzka, Jakub Fichna

**Affiliations:** 1 Department of Biochemistry, Faculty of Medicine, Medical University of Lodz, Mazowiecka 5, 92-215 Lodz, Poland; karolina.merecz@umed.lodz.pl (K.M.); kinga.suska@umed.lodz.pl (K.S.);; 2 Department of Fruit, Vegetable and Plant Nutraceutical Technology, Faculty of Biotechnology and Food Science, Wrocław University of Environmental and Life Sciences, Chelmonskiego 37, 51-630 Wroclaw, Poland; aneta.wojdylo@upwr.edu.pl

**Keywords:** cytokines, inflammation, inflammatory bowel disease, natural products, polyphenols

## Abstract

**Background:** Inflammatory bowel disease (IBD), including Crohn’s disease (CD) and ulcerative colitis (UC), is a group of chronic gastrointestinal (GI) diseases with complex and multifactorial pathophysiology. The global prevalence of IBD is increasing, highlighting the need to develop new therapeutic approaches. Plant-derived extracts have recently gained prominence due to their anti-inflammatory properties. **Methods:** This study investigated: apricot leaves (ALE), peach leaves (PLE), black chokeberry fruit (BCHE), rosehip seeds (RSE), passion fruit seeds (PSE), and linden blossom (LBE) (all at the concentration 10–200 µg/mL) in RAW 264.7 mouse macrophages. Cytotoxicity was assessed using the neutral red uptake (NRU) assay, and anti-inflammatory activity was assessed using Griess assay in the lipopolysaccharide (LPS)-induced inflammation. Additionally, the mRNA expression levels of key inflammatory genes (interferon-γ (*Ifn-γ*), interleukin-6 (*Il-6*), nitric oxide synthase (*Nos2*), and tumor necrosis factor-α (*Tnf-α*)) were analyzed. **Results:** ALE and PLE exhibited minimal cytotoxicity and strong anti-inflammatory activity, reducing the expression of all analyzed genes. PSE demonstrated anti-inflammatory activity in the Griess assay, but did not alter mRNA expression. **Conclusions:** ALE and PLE exhibit promising anti-inflammatory properties and warrant further preclinical investigation. Comprehensive in vitro and in vivo studies are necessary to confirm these results.

## 1. Introduction

Inflammatory Bowel Disease (IBD), which primarily includes ulcerative colitis (UC) and Crohn’s disease (CD), is a chronic inflammatory condition of the gastrointestinal (GI) tract, associated with dysregulated immune responses [1,2,3]. UC manifests as persistent inflammation limited to the mucosal layer of the colon, while CD affects deeper layers of the intestinal wall and is often accompanied by granuloma formation [1,4,5]. Abdominal pain, diarrhea, rectal bleeding, ulceration, malnutrition, and fatigue are among the most common symptoms reported by patients with IBD [1,5]. The etiology of IBD is multifactorial and involves genetic predisposition, environmental and lifestyle factors, dysregulated immune activity, as well as disturbances in the mucosal barrier and/or in the GI microbiota [1,2,3]. Neutrophils, various enzymes, and reactive oxygen species mediate the inflammation leading to disrupted mucosa [6].

The prevalence of IBD has increased rapidly over the past few years. Between 1990 and 2021, the number of IBD cases rose by 88.30% [7]. Globally, the age-standardized incidence rate increased from 4.22 to 4.45 per 100,000 population over the same period, with the most pronounced rise observed among patients aged 50–54 years [7].

Conventional pharmacological treatment of IBD includes aminosalicylates, corticosteroids, immunomodulators, and antibiotics. More recently, biologically driven therapies have also been introduced, including recombinant anti-inflammatory cytokines such as interleukin-10 (IL-10) and interferon-α (IFN-α), as well as monoclonal antibodies targeting pro-inflammatory cytokines (e.g., tumor necrosis factor-α (TNF-α) or interleukin-12 (IL-12)) and their receptors (e.g., TNFR) [1,3,8,9]. Although these therapeutic approaches can alleviate IBD symptoms to varying extents depending on disease stage, their use is frequently associated with adverse side effects [1,2,3].

Plant extracts and herbal medicines have gained increasing attention as promising candidates for new drugs or adjunctive therapeutic approaches for IBD [6,10,11]. They are considered to be less toxic than currently used treatments as their therapeutic potential is mostly linked to a wide range of bioactive compounds, including polyphenols, as well as non-phenolic constituents such as unsaturated fatty acids, vitamins, and dietary fibers [1,10,11,12,13,14].

Polyphenols present in plant extracts may exert anti-inflammatory effects by inhibiting the activation of pro-inflammatory signaling pathways, such as nuclear factor-κβ (NF-κB), and mitogen-activated protein kinase (MAPK), thereby reducing the production of pro-inflammatory cytokines [6,10,15]. At the same time, they enhance antioxidant defenses by increasing the activity of enzymes such as superoxide dismutase and glutathione peroxidase [10]. In addition, polyphenols have been shown to influence gut microbiota composition, reduce inflammation, and improve intestinal barrier integrity, further supporting their therapeutic potential in gastrointestinal disorders [10,16,17].

Although numerous studies have investigated the anti-inflammatory effects of individual plant extracts, herbal medicines, and isolated polyphenols, comparative analyses of chemically diverse plant-derived preparations remain limited [1,10,13,14,15,18,19,20,21,22,23,24,25].

The anti-inflammatory efficacy of dietary polyphenols is strongly influenced by their structural features, including the number and position of hydroxyl groups, the presence of a C2=C3 double bond, and a 4-oxo function in the C-ring of flavonoids, as well as galloylation or polymerization in flavan-3-ols and proanthocyanidins, which modulate redox potential, metal-chelating capacity, and the ability to interfere with key signaling pathways such as NF-κB and MAPK. Quantitative structure-activity relationship (QSAR) studies have shown, for example, that flavones with a C2=C3 double bond and catechol-type substitution in the B ring (e.g., luteolin) display stronger inhibition of NO production, iNOS expression, and pro-inflammatory cytokines in LPS-stimulated RAW 264.7 macrophages than analogues lacking these features, while certain substitutions (e.g., methoxylation at C4′) attenuate activity [26,27]. Similar SAR patterns have been described for other polyphenol classes, supporting the concept that differences in polyphenolic profiles in terms of both qualitative composition and quantitative abundance of specific structural motifs may translate into distinct anti-inflammatory responses and are therefore worth investigating in the context of IBD [27,28].

The selection of different plants and anatomical parts reflects a rational and widely used experimental strategy in polyphenol research, in which chemically diverse, polyphenol-rich plant materials derived from fruits, seeds, flowers, and leaves are tested side-by-side to cover a wide range of phytochemical compositions and to identify the most promising candidates for further preclinical investigation in IBD [17,29,30]. Consequently, the present study aimed to validate the cytotoxicity and anti-inflammatory properties of extracts obtained from apricot leaves (ALE), peach leaves (PLE), black chokeberry fruit (BCHE), rosehip seeds (RSE), passion fruit seeds (PSE), and linden blossom (LBE) in RAW 264.7 mouse macrophages, and to explore their potential relevance for IBD management.

Black chokeberry fruit and rosehip seeds are recognized as rich sources of polyphenols and strong antioxidants, with documented anti-inflammatory and gut-protective effects, which support their inclusion in studies targeting chronic intestinal inflammation and IBD [29,31,32]. Passion fruit seeds have been characterized as valuable by-products, providing high levels of phenolic compounds with significant antioxidant activity, while linden blossom is traditionally used for its anti-inflammatory and soothing properties and contains flavonoids and other phenolics relevant for gastrointestinal health. Fruit seeds generally concentrate flavan-3-ols, phenolic acids, and proanthocyanidins, which confer strong antioxidant and anti-inflammatory potential and modulate key pathways implicated in IBD, such as NF-κB and MAPK signaling, as well as oxidative stress and epithelial barrier integrity [32,33]. Moreover, leaves of stone fruits such as apricot and peach, although less commonly utilized than the edible fruits, are increasingly appreciated as polyphenol-rich plant organs that accumulate high levels of flavonols, flavones, and phenolic acids as part of the plant stress response, and thus represent an underexploited source of bioactive compounds with potential relevance for inflammatory diseases, including IBD [17,31,32].

## 2. Materials and Methods

### 2.1. Extracts Preparation

Homogenous powders from apricot leaves (ALE), peach leaves (PLE), black chokeberry fruit (BCHE), rosehip seeds (RSE), passion fruit seeds (PSE), and linden blossom (LBE) were obtained after 24 h of freeze-drying using Alpha 1-4 LSC freeze-dryer (Martin Christ GmbH, Osterode am Harz, Germany). Extraction was carried out using a polar solvents—water:ethanol (50:50, *v/v*) at a solvent-to-powder ratio of 3:1 (*v/w*). To maximize the recovery of phenolic compounds, the extraction was repeated five times, each following a 1 h maceration period with agitation every 15 min. For the last two extraction steps, the solvent volume was reduced by approximately 30–50%. After each extraction step, solvents were collected and centrifuged at 15,000× *g* for 10 min using a centrifuge (MPW-380R; MPW Med. Instruments, Warsaw, Poland) to remove solid residues. The ethanol fraction was subsequently evaporated under reduced pressure using a rotary evaporator (RV 10; IKA, Königswinter, Germany). The remaining aqueous phase, containing polyphenolic compounds, was applied to a chromatography column packed with Amberlite XAD-16 resin (Sigma-Aldrich; Darmstadt, Germany) preconditioned with water, following the procedure described by Wojdyło et al. [14]. After sample loading, the column was washed with water to remove non-phenolic constituents, and the absorbed phenolic compounds were eluted with 80% ethanol. All fractions were collected, combined, and subjected to ethanol removal at 40 °C using a Hei-VAP Expert (Heidolph; Schwabach, Germany). After ethanol evaporation, the remaining aqueous phase was frozen at −20 °C and subsequently freeze-dried. The resulting polyphenol-rich powdered extracts were used in all further chemical and biological analyses.

### 2.2. Quantitative Analyses of Polyphenols in Tested Extracts

Ultra-high-performance liquid chromatography with a photodiode array detector (UPLC-PDA) was used to identify and quantify polyphenolic compounds in the extracts. Detection was carried out at characteristic wavelengths depending on the chemical group of the compounds: flavan-3-ols—280 nm, flavones and flavonols—360 nm, phenolic acids—320 nm, and anthocyanins—520 nm, following the method described by Wojdyło et al. [14]. Polyphenolic compounds were separated using an ACQUITY UPLC BEH C18 column (1.7 μm, 2.1 × 100 mm; Waters Corporation, Milford, MA, USA) maintained at a constant temperature of 30 °C. The analysis was performed at the following conditions: sample injection volume of 5 μL, total run time 15 min, and a combined gradient/isocratic elution at a flow rate of 0.42 mL/min. The gradient program was initiated with solvent A at 99%, decreasing to 65% over 0–12 min. Subsequently, solvent A was reduced to 0% for the column conditioning (12.5–13.5 min), and then returned to the initial conditions (99% A) by 15 min. The mobile phase consisted of solvent A (water containing 2% and 0.1% formic acid, (*v/v*) and solvent B (100% acetonitrile). All samples were analyzed in triplicate, and the results were reported as mg/g of dry matter with ± SD (Table 1).

The calibration curves were made for the standard (+)-catechin, (-)epi-catechin, and procyanidins B2 for flavan-3-ols, chlorogenic, ferulic, and p-coumaric acid for phenolic acid, luteolin-7-*O*-glucoside and apigenin-7-*O*-glucoside for flavones, quercetin and kaempferol-3-*O*-glucosidase for flavonols, and cyanidin-3-*O*-glucoside for anthocyanins at concentrations ranging from 0.5 to 5 mg/mL and r = 0.999–0.992. Data acquisition and processing were carried out using Empower 3 software (Waters Corp., Milford, MA, USA).

### 2.3. Cell Culture

RAW 264.7 mouse macrophages (ATCC^®^, TIB-71™) were cultured in Dulbecco’s Modified Eagle Medium (DMEM) supplemented with 10% bovine calf serum (BCS), 2 mM alanine-glycine, 0.5% penicillin-streptomycin, 1 mM sodium pyruvate, and 25 mM HEPES buffer. Cells were maintained at 37 °C in a humidified incubator with 5% CO_2_ and 95% humidity.

### 2.4. Neutral Red Uptake Assay

To determine the maximum non-toxic concentrations of the tested extracts, a neutral red uptake (NRU) assay was performed with six technical and three biological replicates. The cells were seeded in 96-well plates at a density of 2 × 10^4^ cells per well. The extracts were added to the selected wells at a final concentration of 200 µg/mL, 100 µg/mL, 50 µg/mL, and 10 µg/mL in a total volume of 200 µL. Wells containing untreated cells served as the control. After 48 h of treatment, 100 μL of neutral red solution (0.05 mg/mL in culture medium) was added to each well, and the plates were incubated for 1 h at 37 °C. The cells were subsequently washed with 200 μL of phosphate-buffered saline (PBS). For dye extraction, 100 μL of eluent solution (composed of 10% acetic acid, 40% ethanol, and distilled water) was added to each well. Plates were shaken for 10 min to ensure complete dissolution of the dye. The reading was conducted using a microplate reader (iMARK Microplate Reader^®^, Biorad^®^, Watford, Hertfordshire, UK) with an absorbance of 540 nm, with the blank control serving as reference. Absorbance values were expressed as percentages and normalized to the untreated cells.

### 2.5. Griess Assay

Nitrate production by RAW 264.7 mouse macrophages was determined using the Griess assay with six technical and three biological replicates. Cells were seeded in 96-well plates at a density of 2 × 10^4^ cells per well. Extracts were added to the wells at a final concentration of 200 µg/mL, 100 µg/mL, 50 µg/mL, and 10 µg/mL in a total volume of 200 µL. Lipopolysaccharide (LPS; 1 µg/mL) was added to the respective wells to induce inflammation. Wells containing untreated cells served as a control. After 24 h of incubation, the culture medium was replaced with fresh medium containing the appropriate extract concentrations or left untreated. Following an additional 24 h incubation period, 100 µL of supernatant from each well was transferred to a new 96-well plates and mixed with 100 µL of Griess reagent. The plates were shaken for 15 min in the dark, and absorbance was measured at 540 nm using a microplate reader (iMARK Microplate Reader^®^, Biorad^®^, Watford, Hertfordshire, UK). Culture medium alone served as the blank reference. Absorbance values were expressed as percentages and normalized to LPS-treated cells.

### 2.6. RNA Isolation

RNA isolation was performed for 200 µg/mL of apricot leaves extract (ALE), 200 µg/mL of peach leaves extract (PLE), and 50 µg/mL passion fruit seeds extract (PSE) using the Total RNA Mini Plus Kit (A&A Biotechnology^®^, Gdansk, Poland), following the manufacturer’s protocol. RAW 264.7 cells were seeded in 6-well plates at a density of 4 × 10^5^ cells per well and treated with either 1 µg/mL LPS alone or 1 µg/mL LPS in combination with a selected concentration of each extract. RNA isolation was performed with six technical and three biological replicates. After 24 h of incubation, the medium was replaced with a fresh one, and the cells were incubated for an additional 24 h. The supernatants were then removed, and each well was washed with 1 mL of PBS. To lyse the cells, 400 µL of Fenozol^®^ Plus was added to each well. Following mixing, samples were incubated for 5 min and centrifuged at 12,000 rpm for 5 min at room temperature. Total RNA was isolated according to the kit protocol. RNA concentration and purity were determined using a Colibri^®^ Microvolume Spectrophotometer (Biocompare, San Francisco, CA, USA).

### 2.7. cDNA Synthesis by Reverse Transcription of RNA & Real-Time RT-PCR

Complementary DNA (cDNA) was synthesized from total RNA using the Maxima First Strand cDNA synthesis Enzyme Kit for RT-qPCR #K1641 (ThermoFisher Scientific™, Waltham, MA, USA) according to the manufacturer’s instructions. Reverse transcription was performed in a Biometra T-Gradient Thermal Cycler (Analytik Jena AG, Jena, Germany) under the following conditions: 25 °C for 10 min, 50 °C for 15 min, 85 °C for 5 min, followed by cooling at 4 °C for 10 min.

Real-time reverse transcription polymerase chain reaction (real-time RT-PCR) reactions were prepared for each gene, TaqMan™ Gene Expression Master Mix, nuclease-free water, and fluorescence-labelled TaqMan probes for the following genes: *Ifn-γ* (Mm01168134_m1), interleukin 6 (*Il-6*; Mm00446190_m1), nitric oxide synthase (*Nos2*; Mm00440502_m1), and *Tnf-α* (Mm00443256_m1). Gene expression levels were normalized to the reference genes *Gapdh* (Mm99999915_g1) or *Actinβ* (Mm00607939_s1). All probes were obtained from ThermoFisher^®^, Waltham, MA, USA.

RT-PCR was performed using Lighcycler^®^ 96 Instrument (Roche Diagnostics, Warsaw, Poland). The cycling conditions were as follows: initial denaturation at 95 °C for 600 s, followed by 50 amplification cycles (95 °C for 10 s and 60 °C for 60 s), and a cooling step at 37 °C for 30 s. All reactions were performed in duplicate. Threshold cycle (Ct) values for the target genes were normalized to the Ct values of the corresponding reference gene. Relative gene expression levels were calculated using the formula: 2^−ΔCt^ × 1000, where ΔCt = Ct (target gene) − Ct (reference gene).

### 2.8. Statistical Analysis

Statistical analysis was performed using GraphPad Prism 8.0.1 (GraphPad Software Inc., La Jolla, CA, USA). Outliers were identified using the Grubbs’ test (α = 0.05). The normality of data distribution was assessed using the Shapiro–Wilk test. Data meeting the assumptions for parametric testing were analyzed using one-way analysis of variance (ANOVA), whereas non-parametric data were analyzed using the Kruskal–Wallis test and Tukey’s post hoc test for polyphenols analysis (*p* < 0.05). Both analyses were followed by Dunn’s post hoc test for multiple comparisons. Data were normalized to baseline values and expressed as percentages (100 × value/baseline). Results are presented as mean ± standard error of the mean (SEM). Differences were considered statistically significant at *p* < 0.05.

## 3. Results

### 3.1. Polyphenolic Profile of the Analyzed Plant Extracts

The total polyphenolic content varied markedly among the analyzed preparations (Table 1), ranging from 14,126.73 to 749,932.5 mg/100 g. The highest levels were observed in PLE, followed by ALE and RSE, whereas PSE showed substantially lower values.

Clear differences were also noted in the distribution of polyphenolic classes. Flavonols dominated in PLE, while ALE exhibited a more balanced profile of flavonols (including quercetin-3-O-rutinoside and kaempferol-3-O-rutinoside) and phenolic acids (such as chlorogenic and gallic acid). In contrast, RSE consisted almost exclusively of phenolic acids. LBE was characterized mainly by flavan-3-ols, with a notable contribution of flavonols.

The most diverse composition was found in BCHE, which contained all analyzed polyphenol classes, including flavan-3-ols, phenolic acids, and anthocyanins. Conversely, PSE, the least abundant sample, was primarily composed of flavones, with only minor amounts of other compounds.

### 3.2. Treatment with Plant-Derived Extracts Affected RAW264.7 Cell Viability Depending on Extract Source and Concentration, as Assessed by the NRU Assay

**Figure 1:**
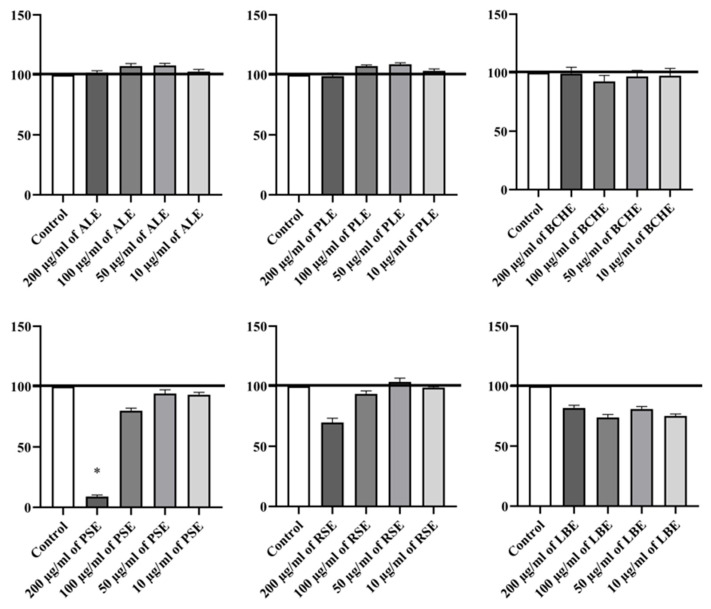
Effects of tested plant extracts on the viability of RAW 264.7 cells based on NRU assay. RAW 264.7 cells were exposed to increasing concentrations of plant extracts (200 µg/mL, 100 µg/mL, 50 µg/mL, and 10 µg/mL) for 48 h. After treatment, cell viability was quantified using the NRU assay. Data were obtained from three independent experiments, with six samples per group. Absorbance values were expressed as percentages and normalized to the non-treated group (control group). Results were presented as mean ± SEM; * *p* < 0.05 vs. control group.

To identify the cytotoxicity of tested plant extracts on RAW 264.7, the NRU assay has been performed. Cells were exposed to ALE, PLE, BCHE, RSE, PSE, and LBE (all at a concentration of 10–200 µg/mL) for 48 h. The viability of RAW 264.7 cells varied depending on the extract used (Figure 1).

Treatment with ALE, PLE, and BCHE across the tested concentrations (10–200 µg/mL) did not significantly affect cell viability, which suggests that these extracts are non-cytotoxic to the cells within a broad concentration range. In contrast, RSE at 200 µg/mL, as well as PSE at 100 and 200 µg/mL, showed cytotoxic effects, reducing cell proliferation compared to the control. Lower concentrations of RSE (10 and 50 µg/mL) and PSE (10 and 50 µg/mL) showed cell viability similar to the control group. Treatment with LBE did not significantly decrease cell viability at all tested concentrations compared to the control. Overall, ALE, PLE, and BCHE were non-cytotoxic across tested concentrations, in contrast RSE, and PSE showed cytotoxicity at higher doses, while LBE showed cytotoxicity at all tested doses.

### 3.3. Treatment with Plant-Derived Extracts Modulated LPS-Induced NO Production in RAW 264.7 Cells Depending on the Concentration of the Tested Extracts, as Assessed by the Griess Assay

LPS-treated RAW 264.7 mouse macrophages were used to evaluate the anti-inflammatory effects of plant extracts (10–200 µg/mL), using the Griess assay. In this test, elevated absorbance values correspond to increased nitric oxide (NO) production, which reflects higher levels of inflammation.

**Figure 2:**
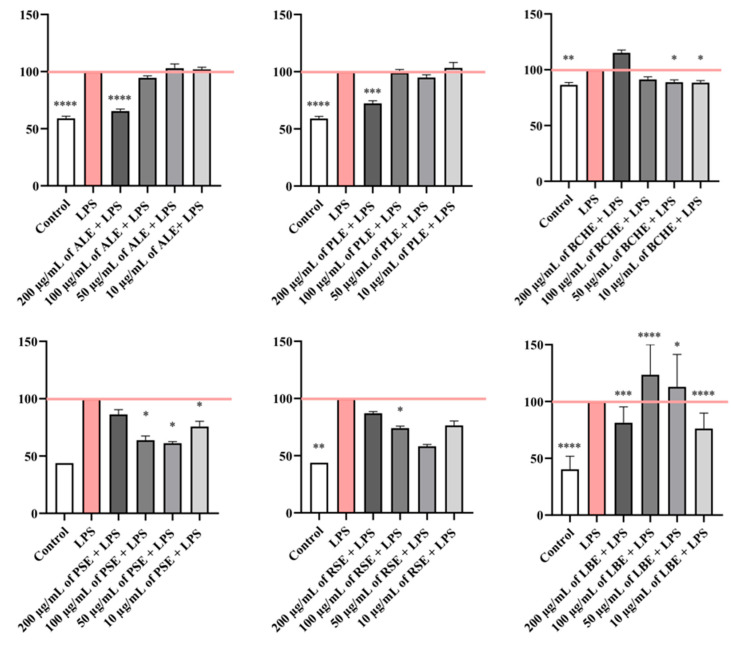
Effects of tested plant extracts on LPS-induced NO production in RAW 264.7 cells based on Griess assay. Cells were exposed to increasing concentrations of plant extracts (200 µg/mL, 100 µg/mL, 50 µg/mL, and 10 µg/mL) and LPS (1 µg/mL) for 24 h, following the addition of Griess reagent. Data were obtained from three independent experiments, with six samples per group. Absorbance values were expressed as percentages and normalized to the LPS-treated group (positive control group). Results were presented as mean ± SEM; * *p* < 0.05, ** *p* < 0.01, *** *p* < 0.001, **** *p* < 0.0001 vs. LPS-treated group. The percentage reduction in NO production compared to the LPS-treated group (set as 100%) for ALE was reduced by 41% in the control group; 34.55% at 200 µg/mL; 5.4% at 100 µg/mL, whereas a slight increase of 2.92% at 50 µg/mL and 1.94% at 10 µg/mL. A reduction in NO for PLE was reduced by 41% in the control group; by 27.66% at 200 µg/mL; 0.8% at 100 µg/mL; 5.08% at 50 µg/mL, whereas a slight increase of 3.30% was observed at 10 µg/mL. For BCHE, a reduction in NO was reduced by 14% in the control group; increased by 15.24% at 200 µg/mL; decreased by 8.71% at 100 µg/mL; by 11.24% at 50 µg/mL; and 12% at 10 µg/mL. A reduction in NO for PSE was reduced by 56.16% in the control group; 13.69% at 200 µg/mL; 36.13% at 100 µg/mL; by 38.82% at 50 µg/mL, and by 24.26% at 10 µg/mL. For RSE, a reduction in NO was reduced by 56.16% in the control group; 12.88% at 200 µg/mL; 26% at 100 µg/mL; by 41.78% at 50 µg/mL, and by 23.61% at 10 µg/mL. For LBE, a reduction in NO production was reduced by 59% in the control group, and by 18.75% at 200 µg/mL; a great increase of 23.61% and 12.96% were observed for in order at 100 µg/mL and 50 µg/mL, while at the smallest concentration, 10 µg/mL, a decrease of 23.84% was observed compared to LPS-treated group.

As shown in Figure 2, incubation of RAW 264.7 mouse macrophages with LPS alone (positive control) induced a significant increase in NO concentration compared to the negative control (non-LPS-treated cells). ALE and PLE, at the highest concentration tested (200 µg/mL), significantly reduced NO production, demonstrating values similar to the negative control group. No significant differences in NO production were observed across the remaining concentrations (10–100 µg/mL) for ALE and PLEs. Treatment with BCHE at lower concentrations (10–50 µg/mL) significantly reduced NO production, while treatment with 200 µg/mL slightly increased induced NO production compared to the LPS-treated group, which may suggest that this extract at higher concentrations may lead to loss of efficacy in the reduction in NO (Figure 2). For RSE and PSE, all tested concentrations decreased the NO production compared to cells treated with LPS alone. The anti-inflammatory effect for 10–100 µg/mL of PSE was statistically significant. Finally, treatment with LBE at 200 µg/mL and 10 µg/mL significantly decreased NO levels, suggesting an anti-inflammatory effect, whereas 100 µg/mL and 50 µg/mL LBE induced an increase in NO concentration, suggesting a possible pro-inflammatory response. However, due to the relatively high variability of the results represented by error bars, the effect of LBE at all testes concentrations on LPS-treated cells could not be clearly determined.

### 3.4. Treatment with ALE and PLE Reduced the Expression of Genes Involved in the Inflammatory Response, as Determined by Real-Time RT-PCR

Based on the NRU and Griess assay results, only ALE (at the concentration of 200 µg/mL), PLE (200 µg/mL), and PSE (50 µg/mL) were selected for further studies. These extracts were chosen as they demonstrated the lowest cytotoxicity and the most effective reduction in NO secretion in LPS-stimulated RAW 264.7 mouse macrophages. Real-time RT-PCR experiments were carried out in two independent series: one including ALE and PLE, and one including PSE.

**Figure 3:**
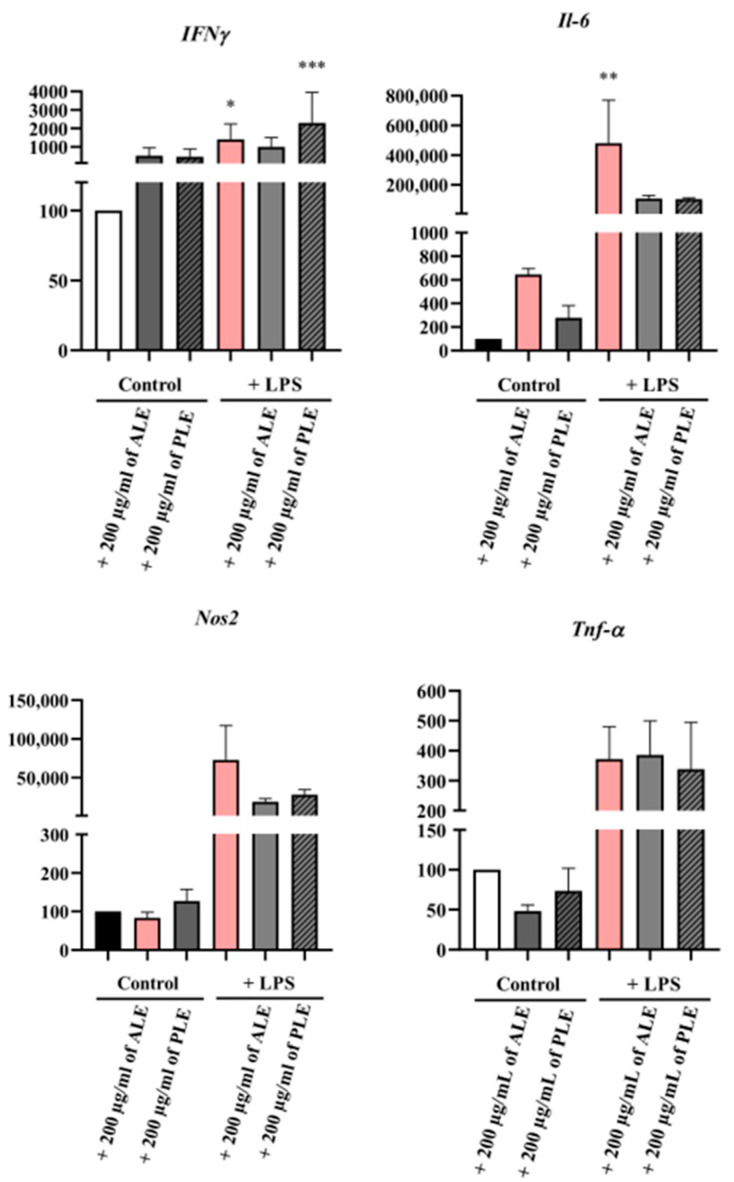
Real-time RT-PCR analysis of *Ifn-γ, Il-6, Nos2*, and *Tnf-α* expression in RAW 264.7 mouse macrophages treated with ALE or PLE (200 µg/mL). Cells were exposed to ALE or PLE (200 µg/mL) and LPS (1 µg/mL) for 24 h, followed by a medium change and further incubation for an additional 72 h. Cells were lysed with Fenozol^®^ Plus. cDNA was synthesized from total RNA and used as a template for real-time RT-PCR. Results were presented as mean ± SEM; * *p* < 0.1, ** *p* < 0.01, *** *p* < 0.0005 vs. control group.

As shown in Figure 3, 200 µg/mL of ALE or PLE treatment did not significantly change *Ifn-γ* expression compared to the negative control (non-LPS-treated cells). Treatment with LPS alone significantly increased *Ifn-γ* expression compared to the non-treated cells. For cells treated with 200 µg/mL of ALE + LPS, the *Ifn-γ* expression level was lower compared to the LPS-treated cells and higher when compared to the control group. Notably, 200 µg/mL of PLE + LPS significantly increased the *Ifn-γ* expression level compared to the LPS group.

Real-time RT-PCR analysis of *Il-6* and *Nos2* displayed similar results (Figure 3). In the absence of LPS, neither ALE nor PLE displayed any significant change in gene expression, comparable to the negative control group, which indicates that treatment with these extracts alone does not stimulate any inflammatory responses. As expected, stimulation with LPS increased the expression of *Il-6* and *Nos2*, reflecting its role in inducing an inflammatory response. ALE or PLE (200 µg/mL) significantly reduced *Il-6* and *Nos2* expression in LPS-stimulated cells compared to LPS alone.

ALE or PLE (200 µg/mL) significantly reduced *Tnf-α* expression in RAW 264.7 cells compared to negative control group. Treatment with LPS alone showed a significant increase in *Tnf-α* expression, in comparison to the negative control group, indicating LPS-induced inflammation. Cells treated with 200 µg/mL of ALE or PLE in combination with LPS expressed *Tnf-α* at levels comparable to the LPS alone-treated group.

**Figure 4:**
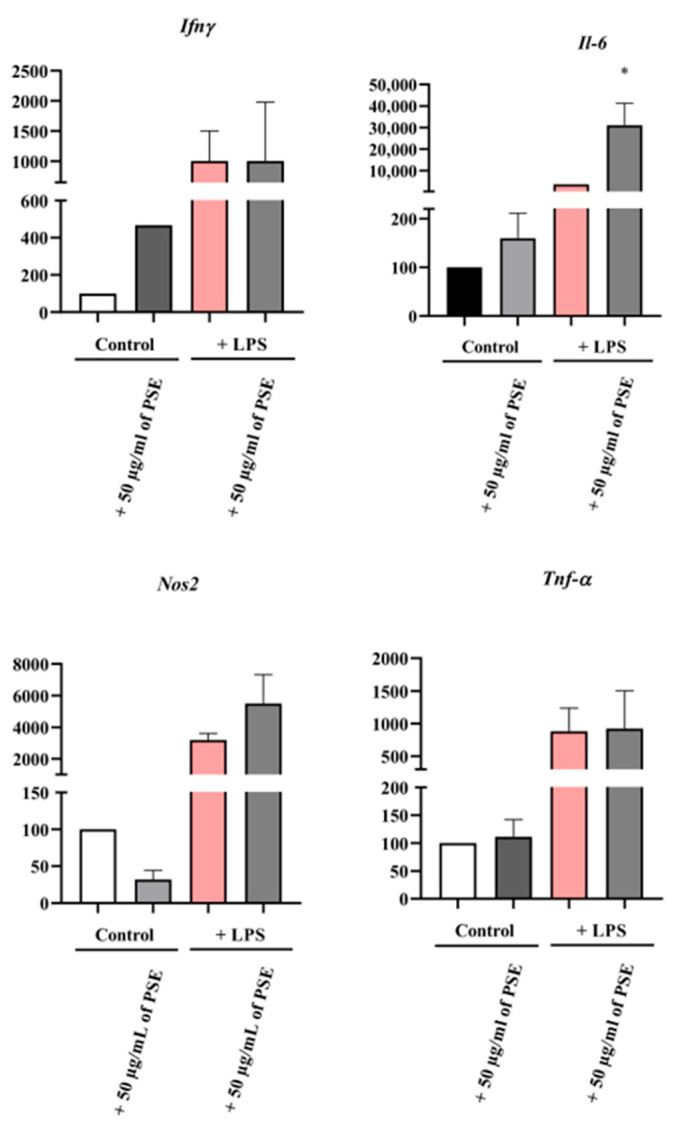
Real-time RT-PCR analysis of *Ifn-γ, Il-6, Nos2*, and *Tnf-α* expression in RAW 264.7 mouse macrophages treated with PSE (50 µg/mL). Cells were exposed to PSE (50 µg/mL) and LPS (1 µg/mL) for 24 h, followed by a medium change and further incubation for an additional 72 h. Cells were lysed with Fenozol^®^ Plus. cDNA was synthesized from total RNA and used as a template for real-time RT-PCR. Results were presented as mean ± SEM; * *p* < 0.1 vs. control group.

As shown in Figure 4, PSE alone increased the *Ifn-γ* expression level compared to the negative control group. Treatment with LPS did not significantly increase *Ifn-γ* expression compared to the negative control, while 50 µg/mL of PSE + LPS exhibited similar results to the LPS-treated cells.

PSE alone had no significant effect on *Il-6* and *Nos2* expression compared to the negative control group. LPS-treated cells did not significantly change the *Il-6* and *Nos2* expression levels when compared to the positive control group. Consequently, an increase was observed in mRNA expression for the cells treated with 50 µg/mL of PSE + LPS compared to the LPS-treated group (Figure 4).

Cells treated with 50 µg/mL PSE indicated similar *Tnf-α* expression levels to those in the negative control group. In cells treated with LPS, no significant increase in *Tnf-α* gene expression was observed compared to the positive control group. Cells treated with 50 µg/mL of PSE + LPS showed results similar to those of the LPS alone-treated cells.

## 4. Discussion

Extracts obtained from various parts of apricot and peach plants, including fruits, kernels, and seeds, exhibit anti-inflammatory potential due to their high content of bioactive compounds such as procyanidins, phenolic acids, flavonoids, catechins, and other polyphenols [14,19,20,34,35,36,37,38]. Concurrently, passion fruit is also rich in biologically active compounds, such as polyphenols (anthocyanins and phenolic acids), vitamin C, and carotenoids, and thus its mesocarp, epicarp, and seeds have been studied for their anti-inflammatory properties in IBD treatment [21].

Polyphenolic compounds have antioxidant properties, including elimination of free radicals and reactive oxygen species, and protection against lipid peroxidation [13]. Phenolic acids are known to lower inflammation, scavenge free radicals, inhibit oxidative stress-induced damage in cells, and act as antioxidant compounds against DNA oxidative changes [13,35,38,39]. In the meta-analysis by Fang Liu et al. stated that given the very complex onset of IBD, different groups of polyphenols might have diverse effects on IBD development and treatment [10].

The present study evaluated the cytotoxicity and anti-inflammatory properties of extracts obtained from apricot leaves (ALE), peach leaves (PLE), and passion fruit seeds (PSE) in RAW 264.7 macrophages. Their potential relevance for IBD management was also investigated. ALE and PLE showed no cytotoxicity in the NRU assay and demonstrated strong anti-inflammatory activity in the Griess assay. In contrast, PSE reduced nitric oxide production but showed cytotoxic effects at higher concentrations. Moreover, the treatment with ALE and PLE decreased the expression of all analyzed genes: *Ifn-γ*, *Il-6*, *Tnf-α*, and *Nos2* in LPS-treated macrophages, further validating the anti-inflammatory potential of these extracts. In contrast, treatment with PSE did not influence the expression of one analyzed pro-inflammatory cytokine (*Ifn-γ*), and increased the expression of other analyzed genes (*Il-6*, *Tnf-α*, and *Nos2*). IFN-γ, IL-6, and TNF-α, along with NOS2, are key regulators of immune responses and play essential roles in the maintenance of immune homeostasis.

Increased expression of IFN-γ is associated with excessive immune responses and mucosal damage [40]. This cytokine regulates pro-inflammatory genes and disrupts intestinal permeability. It also impairs epithelial and vascular barriers in the gut, contributing to IBD pathology [40]. In the study carried out by Singh et al., the serum samples from 42 patients with UC and CD, as well as healthy donors were examined [41]. Samples from IBD patients were characterized by much higher IFN-γ levels compared to the control group (healthy patients) [41].

Chuo et al. tested various concentrations (50, 100, 200, 500, 800, and 1000 µg/mL) and drying methods (vacuum freeze drying, natural drying, and hot air drying) of polyphenol extracts of thinned peach (PETP) [34]. The researchers investigated the immunomodulatory activity of PETP in LPS-treated RAW 264.7 cells and the involvement of the NF-κβ signaling pathway [34]. The study demonstrated that LPS activated the NF-κβ signaling pathway and modulated different cytokines such as IFN-γ [34]. Noteworthy, 50 µg/mL PETP enhanced immune responses in macrophages, while 800 µg/mL of PETP inhibited the development of inflammation [34]. In line with our study, LPS treatment significantly increased *Ifn-y* expression in RAW 264.7 cells compared to the control group. Incubation with 200 µg/mL of ALE decreased *Ifn-γ* expression compared to the cells treated with LPS alone, but the *Ifn-γ* expression did not significantly change compared to the control group. In contrast, 200 µg/mL of PLE significantly increased *Ifn-γ* expression compared to both LPS-treated and control groups. Incubation with 50 µg/mL of PSE did not significantly increase *Ifn-γ* expression compared to LPS-treated cells and control group of non-treated cells.

IL-6 is another cytokine with a pleiotropic effect on inflammation as it stimulates the expression of various pro-inflammatory cytokines, such as IL-1β and TNF-α [42,43]. An impaired IL-6 production is commonly associated with inflammatory states, including CD and UC, and increased IL-6 serum levels have been observed in both acute and chronic inflammation [44]. In the study by Hyams et al., a strong correlation between IL-6 levels and acute phase proteins was found in children with UC and CD [44]. Moreover, the study reported that higher IL-6 concentrations were associated with more severe disease [44].

The previously mentioned experiment conducted by Chuo et al. demonstrated that LPS modulated various inflammatory cytokines, including IL-6 [34]. Additionally, 50 µg/mL of PETP increased immune responses in this setup [34]. Chusongdam et al. treated RAW 264.7 cells with LPS and PSE (10–100 µg/mL), observing a significant decrease in IL-6 expression, which confirmed PSE’s anti-inflammatory potential [45]. In the study by Lopes do Carmo et al., a co-culture of Caco-2 and RAW 264.7 mouse macrophages was used [46]. The cells were treated with LPS and passion fruit leaves extract [46]. Treatment with passion fruit leaves extract inhibited the release of pro-inflammatory cytokines such as IL-6 and preserved the intestinal barrier function by downregulating paracellular permeability [46]. Similarly, in our study, a significant increase in *Il-6* expression was observed in LPS-treated cells compared to the control group. Incubation with ALE and PLE reduced *Il-6* expression levels compared with the LPS-alone group, which may indicate their anti-inflammatory potential. For the cells treated with 50 µg/mL of PSE + LPS, a significant increase was observed in *Il-6* expression compared to the LPS-treated group.

TNF-α stimulates the production of other pro-inflammatory molecules, such as IL-1β and IL-6, and impairs intestinal epithelial tissue [1,47]. Singh et al. observed that TNF-α levels were overexpressed in IBD patients when compared to the control group (healthy patients) [41].

The study by Chuo et al. evidenced that PETP modulated pro-inflammatory cytokines, including TNF-α, in LPS-treated RAW 264.7 macrophages [34]. Concurrently, Chusongdam et al. demonstrated that PSE significantly suppressed the secretion of TNF-α when compared to the LPS-treated group [45]. In our study, treatment with LPS increased *Tnf-α* levels compared to the control. Moreover, 200 µg/mL of ALE did not significantly increase *Tnf-α* expression compared to LPS-only group, while 200 µg/mL of PLE decreased *Tnf-α* expression level compared to LPS-only group. PSE exhibited results similar to ALE, as incubation with 50 µg/mL of PSE slightly increased *Tnf-α* gene expression level compared to the LPS-group.

The NOS2 gene encodes inducible nitric oxide synthase (iNOS), which contributes to nitric oxide (NO)-mediated tissue damage in IBD pathology [48,49]. Kimura et al. reported significantly increased iNOS activity and NO production in colonic mucosa from patients with active CD or UC, confirming the role of NO in intestinal inflammation [50]. Koyu et al. evaluated cytotoxic, antimicrobial, and NO-inhibitory activities of supercritical carbon dioxide extracts from peach leaves [49]. Multiple cell types, including RAW 264.7 macrophages, were used to determine the effects of PLE on iNOS expression in an LPS-induced inflammation model. The extract suppressed NO production [49]. Similarly, Chusongdam et al. showed that PSE reduced NO production in LPS-stimulated RAW 264.7 cells by downregulating iNOS expression [45], whereas Lopes do Carmo et al. found that passion fruit leaves extract did not reduce NO in a co-culture of Caco-2 and RAW 264.7 cells treated with LPS [46]. In our study, LPS treatment significantly increased *Nos2* expression compared to untreated controls. ALE and PLE attenuated this increase, although expression remained above baseline. In contrast, PSE further elevated *Nos2* levels compared to the LPS-only group.

Several factors should be considered when interpreting these results. Future studies could examine a broader range of extract concentrations and additional plant sources containing similar bioactive compounds. Expanding the panel of pro-inflammatory genes, for example, IL-1β, IL-8, IL-17, IL-18, and IL-23, would provide a more comprehensive understanding of anti-inflammatory responses. In addition, future studies should also focus on anti-inflammatory markers, including IL-10 and TGF-β, to improve insight into the regulation of immune cell recruitment and activation. Moreover, analyses at the protein level using Western blot or ELISA are recommended to validate RNA-level findings. Future work should also focus on identifying the molecular mechanisms, including signaling pathways such as NF-κB, and MAPK, and their potential interactions with oxidative stress pathways and epithelial barrier function. Future studies could include additional in vitro studies integrating other cell types, such as Caco-2, human colon cancer-derived cell line (HT29), and T84 cells, to explore potential differences in gene expression profiles compared to RAW 264.7 cells. The use of advanced systems as co-culture models and organoids cultured with macrophages and lymphocytes might allow for a better modelling of mucosal function.

While ALE and PLE demonstrate promising anti-inflammatory activity in vitro, in vivo studies are necessary for a better understanding of the complexity of the intestinal microenvironment and to confirm the therapeutic potential and safety of the plant-derived extracts. In vivo studies using relevant animal models of IBD are necessary to validate the observed anti-inflammatory effects, assess bioavailability, pharmacokinetics, and evaluate safety profiles before considering ALE and PLE as potential adjunctive treatments for IBD.

## 5. Conclusions

The results of this study indicate that plant-derived extracts from ALE and PLE exhibit promising anti-inflammatory properties in LPS-stimulated RAW 264.7 macrophages. Importantly, both extracts showed no cytotoxic effects within the tested concentration range. These findings suggest that ALE and PLE may represent potential natural candidates for the development of supportive therapeutic strategies in IBD. Nevertheless, further studies using additional in vitro models and in vivo systems are required to confirm their biological activity and evaluate their clinical relevance.

## Figures and Tables

**Table 1:** Content of individual polyphenol classes in selected plant extracts (mg/g dry matter).

Polyphenol Classes	ALE	PLE	BCHE	RSE	PSE	LBE
Flavan-3-ols	nd	nd	66.4 ± 2.43	nd	10.17 ± 0.54	127.84 ± 5.71
Phenolic acids	223.45 ± 14.56	34.75 ± 2.65	61.90 ± 1.88	437.86 ± 15.54	4.09 ± 0.93	3.93 ± 0.21
Flavonols	240.19 ± 10.32	715.18 ± 15.53	3.90 ± 0.43	1.37 ± 0.32	23.24 ± 1.34	81.42 ± 3.66
Flavones	nd	nd	0.90 ± 0.12	nd	73.41 ± 4.29	nd
Anthocyanins	nd	nd	51.90 ± 2.07	nd	30.35 ± 7.17	nd
In total	463.64 ^b^	749.93 ^a^	185.00 ^d^	439.24 ^b^	141.27 ^e^	213.18 ^c^

nd—not detected; ALE, apricot leaves; PLE, peach leaves; BCHE, black chokeberry fruit; RSE, rosehip seeds; PSE, passion fruit seeds; LBE, linden blossom. Values are expressed as mean ± standard deviation (SD). Different letters within the same row indicate statistically significant differences between samples (*p* < 0.05), as determined by one-way ANOVA followed by Tukey’s post hoc test.

## Data Availability

Data are contained within the article.

## Abbreviations

The following abbreviations are used in this manuscript:

ALE	apricot leaves extract
Caco-2	cancer-coli-2
CD	Crohn’s disease
CSs	corticosteroids
COX-2	cyclooxygenase-2
GAPDH	glyceraldehyde-3-phosphate dehydrogenase
GI	gastrointestinal
iNOS	inducible nitric oxide synthase
IBD	inflammatory bowel disease
IFN-γ	interferon-γ
IL	interleukin
LPS	lipopolysaccharide
MAPK	mitogen-activated protein kinase
NF-κβ	nuclear factor-kappa β
NOS2	nitric oxide synthase
NRU	neutral red uptake
PLE	peach leaves extract
ROS	reactive oxygen species
TNF-α	tumor necrosis factor-α
UC	ulcerative colitis
UPLC-PDA	ultra-high performance liquid chromatography with a photodiode detector
